# An optimizing method for performance and resource utilization in quantum machine learning circuits

**DOI:** 10.1038/s41598-022-20375-5

**Published:** 2022-10-10

**Authors:** Tahereh Salehi, Mariam Zomorodi, Pawel Plawiak, Mina Abbaszade, Vahid Salari

**Affiliations:** 1 Department of Computer Engineering, Ferdowsi University of Mashhad, Mashhad, Iran; 2 Department of Computer Science, Faculty of Computer Science and Telecommunications, Cracow University of Technology, Krakow, Poland; 3 Institute of Theoretical and Applied Informatics, Polish Academy of Sciences, Gliwice, Poland; 4 Department of Physics, Isfahan University of Technology, Isfahan, 84156-83111 Iran; 5 Institute for Quantum Science and Technology, and Department of Physics and Astronomy, University of Calgary, Calgary, T2N 1N4, Alberta, Canada; 6 Basque Center for Applied Mathematics (BCAM), Bilbao, Spain

**Keywords:** Quantum information, Quantum simulation, Theoretical physics

## Abstract

Quantum computing is a new and advanced topic that refers to calculations based on the principles of quantum mechanics. It makes certain kinds of problems be solved easier compared to classical computers. This advantage of quantum computing can be used to implement many existing problems in different fields incredibly effectively. One important field that quantum computing has shown great results in machine learning. Until now, many different quantum algorithms have been presented to perform different machine learning approaches. In some special cases, the execution time of these quantum algorithms will be reduced exponentially compared to the classical ones. But at the same time, with increasing data volume and computation time, taking care of systems to prevent unwanted interactions with the environment can be a daunting task and since these algorithms work on machine learning problems, which usually includes big data, their implementation is very costly in terms of quantum resources. Here, in this paper, we have proposed an approach to reduce the cost of quantum circuits and to optimize quantum machine learning circuits in particular. To reduce the number of resources used, in this paper an approach including different optimization algorithms is considered. Our approach is used to optimize quantum machine learning algorithms for big data. In this case, the optimized circuits run quantum machine learning algorithms in less time than the original ones and by preserving the original functionality. Our approach improves the number of quantum gates by 10.7% and 14.9% in different circuits respectively. This is the amount of reduction for one iteration of a given sub-circuit U in the main circuit. For cases where this sub-circuit is repeated more times in the main circuit, the optimization rate is increased. Therefore, by applying the proposed method to circuits with big data, both cost and performance are improved.

## Introduction

In recent years, the phenomenon of quantum computing has received global attention^1^. Quantum computational theory goes back to the works by Feynman and Deutsch in the 1980s^2^ and after that many new quantum computing algorithms have been proposed. Machine learning is the science and art of building computers to learn from data how to solve problems instead of explicitly programming. So, Machine learning and quantum computing are two very important research areas, and by combining these two areas, new solutions for today’s challenges are proposed^3^. There are some challenges for implementing quantum machine learning algorithms due to the processing of large datasets. One usual way to meet these challenges is to arrange these algorithms in the cloud system. With the help of the computing power of the cloud system, the problems are partially solved. However, data storage and management in heterogeneous distributed networks had a number of other problems. Physicists take a different method of quantum computing by exploiting the “Superposition” and “Entanglement” properties^9^. This solution has particularly increased the speed of solving certain problems compared to classical algorithms^4,5^. Problem-solving is very different in quantum and classical systems. In fact, some problems that can be solved in the classical system in several years, it is known that can be solved in a quantum system in a few hours^6^. On the other hand, in recent years many types of research have been carried out on the subject of big data. The challenge is the inefficiency of the computations of classical machine learning algorithms and metaheuristics for processing such a large volume of data^7–9^. The unit of quantum processing is the “quantum bit” or “qubit”. One of the capabilities of a quantum computer is that by increasing the number of qubits of a quantum computer, the processing power improves exponentially^10^. Quantum algorithms usually express computations by primitive quantum gates. There are different approaches to implementing these algorithms. Therefore, it is useful to find an implementation using the least resource numbers, especially for large-scale quantum circuits with complex designs. To this end, we apply optimization methods which is a fundamental task in almost all areas of quantum computing science, including monolithic and distributed quantum circuits ^10–13^. This work has developed and implemented a framework for quantum circuit optimization algorithms to optimize the desired circuits which are designed particularly for machine learning tasks. We also show how to optimize the repetition of quantum circuits and reduce the required resources for large-scale quantum circuits. While the original functionality of the algorithm is preserved, the final quantum circuit has fewer time steps, execution time, and quantum cost compared to the original circuit. As input, we assumed that the quantum circuit (QC) consists of a set of quantum gates with a certain number of 2 qubits . The ultimate goal of optimizing the quantum circuit of a machine learning algorithm is to reduce the number of gates, time steps, and quantum cost. The quantum cost of a circuit is the number of 1$\times$1 and 2$\times$2 quantum gates in its design^14^. For this purpose, this paper proposes a method to optimize the quantum cost of machine learning algorithms. In principle, it can be said that the operations involved in quantum machine learning circuits can be large and so it is worth reducing them. Quantum circuits typically use single-qubit and two-qubit gates such as NOT, Hadamard, and rotation, and also two-qubit CNOT gates. If there are three-qubit gates such as Bridge, and Swap, and multi-qubit gates, we decomposed them into single-qubit and two-qubit gates in a preprocessing step. In “Quantum gates and circuits”, we discussed related work in the field of the quantum computation systems for the machine learning algorithm, as well as optimization algorithms for the quantum circuits. Then, in “Quantum memory”, the proposed method is explained and at the end, in “Related work” and “Quantum circuits optimization techniques”, our results and discussion are presented and we conclude the paper.

## Quantum gates and circuits

**Figure 1:**
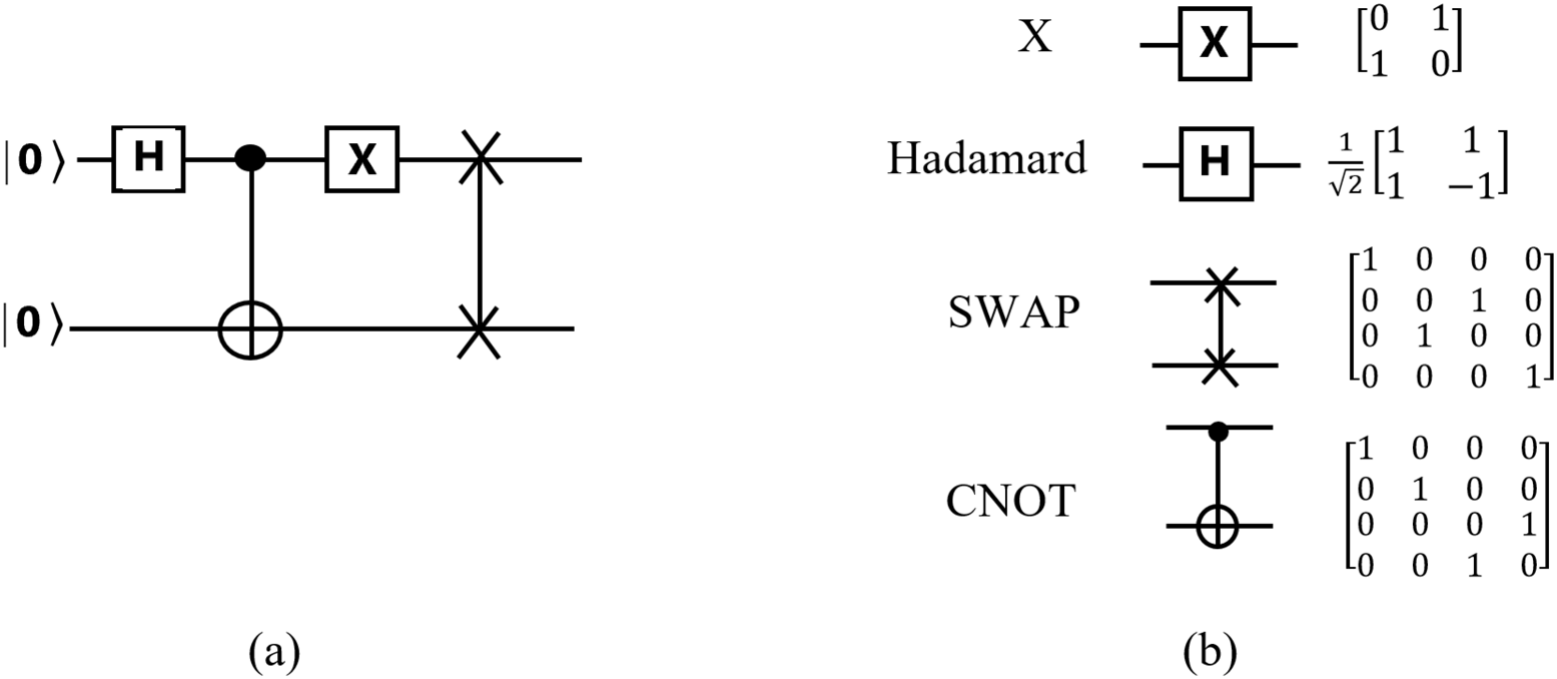
(**a**) An example for a quantum circuit: each line represents a qubit, and operations (gates) are applied on them. (**b**) Representation of unitary two-qubit gates in the quantum circuit with matrix formalism^17^.

Quantum circuit is made up of a combination of Von Neumann and classical architecture^15^, which is executed in the quantum processor. Quantum circuits are shown in such a way that the desired gates are on vertical lines, each of which represents a basic operation. The running time is calculated in these circuits from left to right^16^ using time steps of the quantum circuit. Quantum gates can be represented as $2^n$ dimensional matrices that contain the amplitude of the fundamental states $2^n$ of an n-dimensional quantum system. Figure 1 shows some examples of quantum circuits and their corresponding gates in matrix representation. Figure 1a shows a sample quantum circuit, and Fig. 1b represents the unitary two-qubit gates with their corresponding matrices^17^. Time advances from left to right.

## Quantum memory

The memory of a classic computer can be easily built by writing an arbitrary bit string in any position. Classical memory performance is not optimal for processing big data. In order to solve the problem of normal and associative memory capacity, quantum memory has been used successfully. In many applications of quantum computers, a quantum register is used instead of classical memory to simulate a physical system. This quantum memory consists of a qubit state tensor in multidimensional Hilbert space that is first prepared in a simple state. For a quantum memory consisting of two qubits $|q_0\rangle$ and $|q_1\rangle$, its state $|q_R\rangle$ is given by Eq. (1). The symbol $\otimes$ is the tensor operation. For example, for a 4-qubit quantum register, its state is represented as Eq. (2), where the probability of measuring each of its base states is as different four terms illustrated in Eq. (3)^18,19^. Giovannetti et al.^20^ have demonstrated that how classical data is presented in the language of quantum mechanics through the study of quantum random access memory (QRAM).

1 $$\begin{aligned}&|q_R \rangle = |q_0 \rangle \otimes |q_1\rangle = |q_0 \rangle |q_1\rangle = |q_0 q_1\rangle \end{aligned}$$

2 $$\begin{aligned}&|q_R \rangle = c_0|00 \rangle + c_1|01\rangle + c_2|10\rangle + c_3|11\rangle \end{aligned}$$

3 $$\begin{aligned}&|c_0|^2 + |c_1|^2 + |c_2|^2 + |c_3|^2 = 1 \end{aligned}$$

Implementing machine learning algorithms in quantum computing has two advantages: storage scale and high execution speed of algorithm^9^. By exploiting the superposition property, it is possible that quantum storage be reduced exponentially. According to Eqs. (4) and (5), all binary numbers from the set $\{0,1,\ldots ,i ,\ldots , 2^n-1\}$ are placed in n qubit quantum memory with probability $|c_i|^2$ for state $|i \rangle$.

4 $$\begin{aligned} |\phi _1 \phi _2 \cdots \phi _n \rangle = \sum _{i=0}^{2^n-1} c_i |i \rangle \end{aligned}$$

where

5 $$\begin{aligned} \sum _{i=0}^{2^n-1}|c_i|^2 = 1 \end{aligned}$$

In the classical system for Eq. (6), the operation must be repeated $2^n$ times. But in the quantum system, the system can examine all computational states for the variables simultaneously, assuming that the $U_f$ operator understands the function *f*(*x*)^9^.

6 $$\begin{aligned} U_f\left( \dfrac{1}{\sqrt{2^n}} \sum _{x=0}^{2^n-1}|x \rangle \right) = \dfrac{1}{\sqrt{2^n}} \sum _{x=0}^{2^n-1}U_f|x \rangle = \dfrac{1}{\sqrt{2^n}} \sum _{x=0}^{2^n-1}|f(x) \rangle \end{aligned}$$

## Related work

In this section, we first present some works done in implementing a quantum circuit for quantum machine learning algorithms and then methods for quantum circuit optimization are presented.

### Quantum circuits of machine learning algorithms

Recently, quantum machine learning is considered as a suitable solution to increase the speed of execution of algorithms. This method has led to the introduction of various quantum algorithms for machine learning using quantum features. In this paper, we first examine the K nearest neighbor algorithm. In this regard, Lioyd et al.^21^ and Wiebe et al.^22^ use similar approaches such as quantum amplitude estimation or Grover algorithm^23^ to obtain the quantum state of the nearest neighbor algorithm. In the next method for implementing the nearest neighbor algorithm, Buhrman et al.^24^ use quantum parallelism and the test circuit to calculate the distance between two vectors and provide a quantum solution. The Euclidean distance can be calculated as Euclidean distance$=\sqrt{((2-2|\langle x|y\rangle |))}$. The next method in K nearest neighbor by Ruan et al.^25^ is used in document classification, image classification, etc. It works based on the size of the Hamming distance. A natural vector is defined as a bit vector with a hash function and then converted to an equivalent quantum state, after that, then the input vector bits are compared with the training vector. The number of different properties is counted by the Kaye circuit^25^ and the distance between the two vectors is estimated. The next algorithm, the support vector machine (SVM), is a supervised algorithm developed by Arodz and Saeedi^26^ and also by Rebentrost et al.^27^, which classifies vectors in a specific space based on training data. In comparison with the classical support vector machine for binary classification, they achieved a logarithmic acceleration. These methods use Grover algorithm and adiabatic algorithm. The next algorithm is the neural network algorithm. Transfer learning is an interesting technique in neural networks in which a pre-trained model is reused as an input model for a new task. One of the works in quantum neural networks presented in this field was developed by Acar et al^28^ and uses the quantum transfer learning method. This method is a hybrid machine learning method consisting of a classical network feature extractors and a diverse quantum classification circuit. There are other works by Zen et al.^29^ that have used transmission learning toward scalable quantum neural network states using transmission learning. A protocol was proposed in^47^ for machine translation based on quantum long short term memory for translating a sentence from English to Persian. In another work, Mishra et al.^30^ used the design and operation of a classical neural network and they designed a quantum neural network capable of working on a 10 qubit system. By demonstrating network performance, they have tried to use the basic principles of machine learning to manage data that can be used in cancer detection.

## Quantum circuits optimization techniques

There are many different optimization methods for quantum circuits that aim to reduce the number of time steps, gates, depth, and more. For this purpose, quantum circuits can be improved using different approaches in circuit diagram models, or using circuit simplification rules. For example, the quantum circuit can be improved by the quantum Karnaugh map method^31^, the exclusive sum of product method^32^ and etc. Using circuit simplification rules, there are many different methods that improve circuits by specific rules. The following is a review of the works in this area. Fan et al.^33^ proposed a quantum approximate optimization algorithm, which is a standard method for combinatorial optimization with a gate-based quantum computer. The paper introduced a new Gibbs objective function and demonstrated its superiority, and used the architecture of an Ansatz search algorithm to search for the discrete space of a quantum circuit. These changes led to the improvement of various circuits. Using this method, the median has been increased to 244.7% and 44.4% for the grid and complete graph models of quantum computation. Median reduction in the number of two-qubit gates is 33.3% and 20.8%, respectively. In another paper, Alam et al.^34^ proposed a method to accelerate the implementation of the quantum approximation optimization algorithm (QAOA). First, a connection is made between the classical optimizer and the quantum computer, and then two parameters named $\delta$ and $\beta$, with initial values of zero, are inserted into the loop. The classical optimizer for randomly defined variables initially set to some random values. If the values are not ideal, it establishes a connection to the quantum computer. increases the depth of the circuit, which is not good and should be reduced. For this reason, to determine the appropriate distance for the parameters, artificial intelligence techniques are used to achieve the desired result with the acceleration in the process. This method shows that the number of optimization iterations can be reduced 44.9% on average for 264 graphs. Haner et al.^15^ optimized the circuit using the Hoare triples^35^. This method checks the accuracy of the execution of specific programs. For each circuit level, a pre-condition defines conditions and post-conditions, and based on the previous level condition, the authors can decide on the operating conditions for the next level operation. When using a Hoare-based optimization strategy, the circuit depth decreases for $n \ge 2$, according to relation $(4(n-2)+n)/n$. In the next method of Childs and Maslov^16^, the automatic optimization of large circuits is accomplished using iterative parameters. This method also preserves the main structure of the algorithm and performs better optimizations than state-of-the-art approaches. In fact, it uses a set of exploratory laws that reduces the number of gates. This technique first displays the quantum circuit as a netlist and then preprocesses and simplifies the circuit. Then, it divides the circuit into sub-circuits and optimizes the sub-circuits according to the rules 1-4. In^36^, Abdessaied et al. used several algorithms to synthesize reversible functions to quantum circuits and to reduce the number of Hadamard gates. This reduction of the Hadamard gate, reduces the number and depth of T gates, which improves the combined gates. By applying this method, the authors improved the number and depth of T gate by 88% more than other optimization methods. One other approach for quantum circuit optimization is based on ZX-calculus which is a graphical language for expressing quantum computation^49^. The optimization approach uses the rules of the ZX-calculus for simplifying ZX- diagrams^50^. The authors show that their simplification procedure works well when there are few non-clifford gates in the original circuit. Using different quantum circuit optimization techniques, the aim of this paper is to improve the performance of quantum machine learning circuits and to reduce their cost. To this end we optimized the quantum machine learning circuits in terms of quantum gates and time steps.

## Methods

Implementing machine learning algorithms with big data in quantum systems is a major challenge due to the excessive increase in the number of gates, the depth of the circuit, and the execution time of the algorithm. Optimizing quantum circuits is an effective way to overcome these problems. In this section, the details of the optimization algorithm for quantum machine learning circuits are explained. This method is then used to optimize the quantum circuits of two machine learning algorithms, transmission learning and neural networks. Initially, in the preprocessing step, the quantum circuit represented as a list of gates that are applied sequentially. The following transformation rules are then applied to optimize the quantum machine learning circuits.

**Figure 2:**
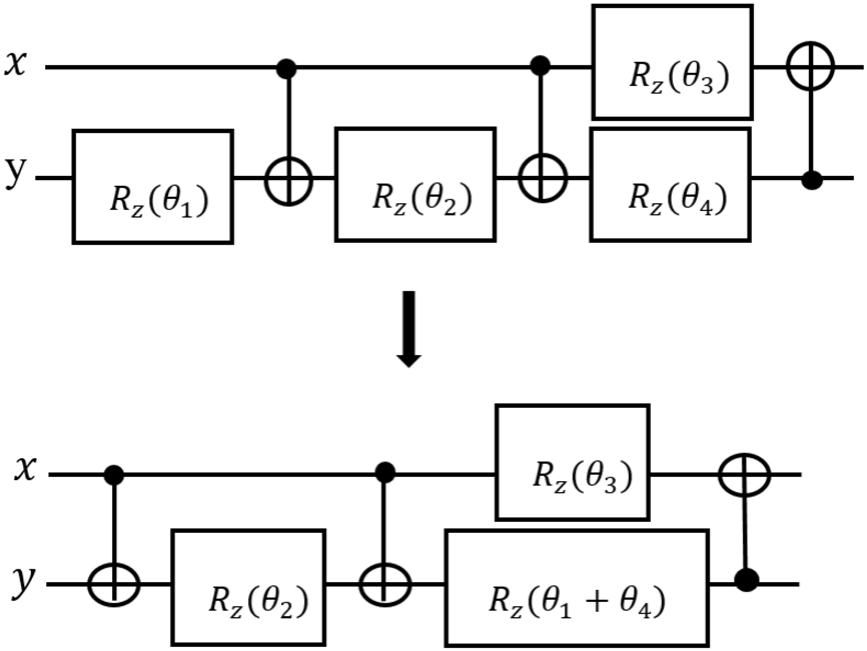
Integration of two rotation gates^16^.

**Rule 1**: First, if there is a NOT gate in the circuit, the next gate is checked. In this case, there are three different possibilities for the next gates^16^:

- If the next gate is a TOFFOLI gate: in this case the control qubit of the TOFFOLI is reversed and the NOT gate is removed.
- If the next gate is a NOT gate: in this case the two NOT gates are removed.
- If the next gate is a CNOT gate: in this case the control qubit is reversed and the NOT gate is removed.

**Rule 2**: Remove gates that are directly adjacent to their inverse. In a two-qubit gate, it is usually possible to simplify or eliminate the gate in the form of quantum circuits by moving it between the gates. In fact, for each U gate in the circuit, the optimizer searches for an instance of $U^{\dagger }$. If present, U is successfully canceled with some instances of $U^{\dagger }$.

**Rule 3**: For two rotation gates $R_Z (\theta _i)$ and $R_Z (\theta _j)$ that have a shared control line, According to Eq. (7), we can merge two rotations^37^. For example, in Fig. 2 two rotation gates $R_Z (\theta _1 )$ and $R_Z (\theta _4)$ can be combined^16^:

7 $$\begin{aligned}&R(\theta _1 )\cdot R(\theta _2 )=R(\theta _1+\theta _2) \end{aligned}$$

8 $$\begin{aligned}&R(\theta _1 )\cdot R(\theta _2)=R(\theta _2)\cdot R(\theta _1) \end{aligned}$$

**Figure 3:**
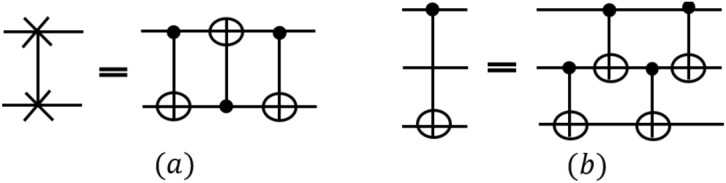
(**a**) Equivalent circuits of Swap gates. (**b**) Equivalent circuits of Bridge gates^38^.

**Rule 4**: Because many quantum algorithms can be described using Swap and Bridge gates^38^, we transform them into the equivalent circuits consisting of CNOT gates on two consecutive qubits. Figure 3a is the equivalent circuit of Swap gate and Fig. 3b is the equivalent circuit of Bridge gate. By breaking down multi-qubit gates into simpler gates, the resulting circuit performs better when using other rules^38^.

**Figure 4:**
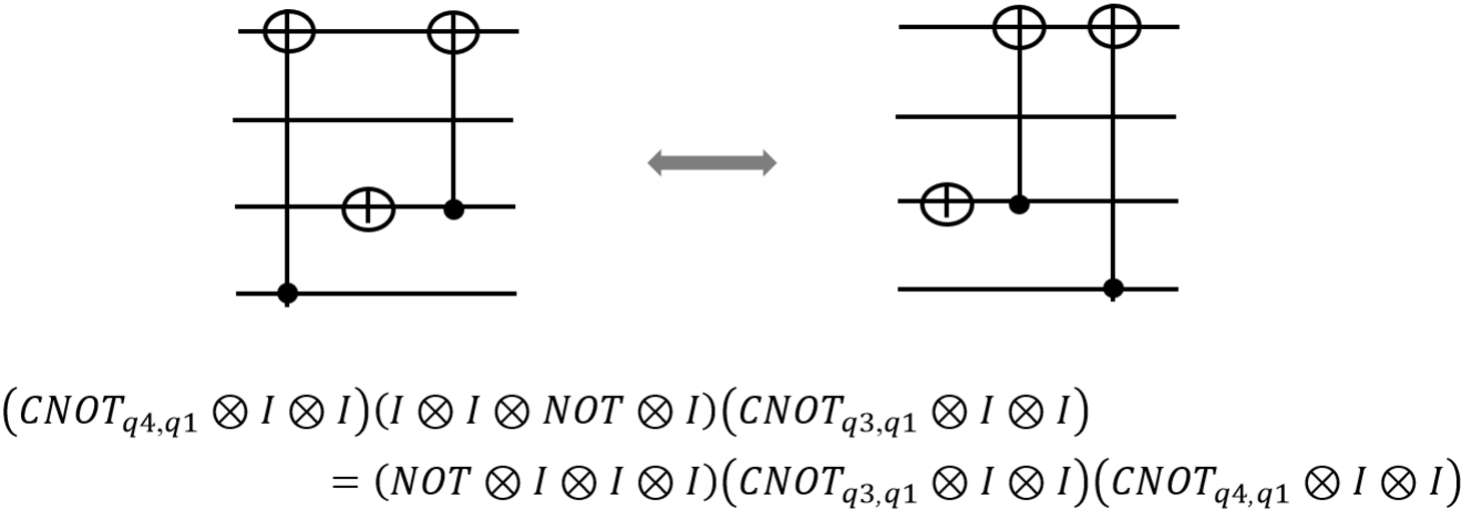
A quantum circuit and its equivalent.

**Figure 5:**
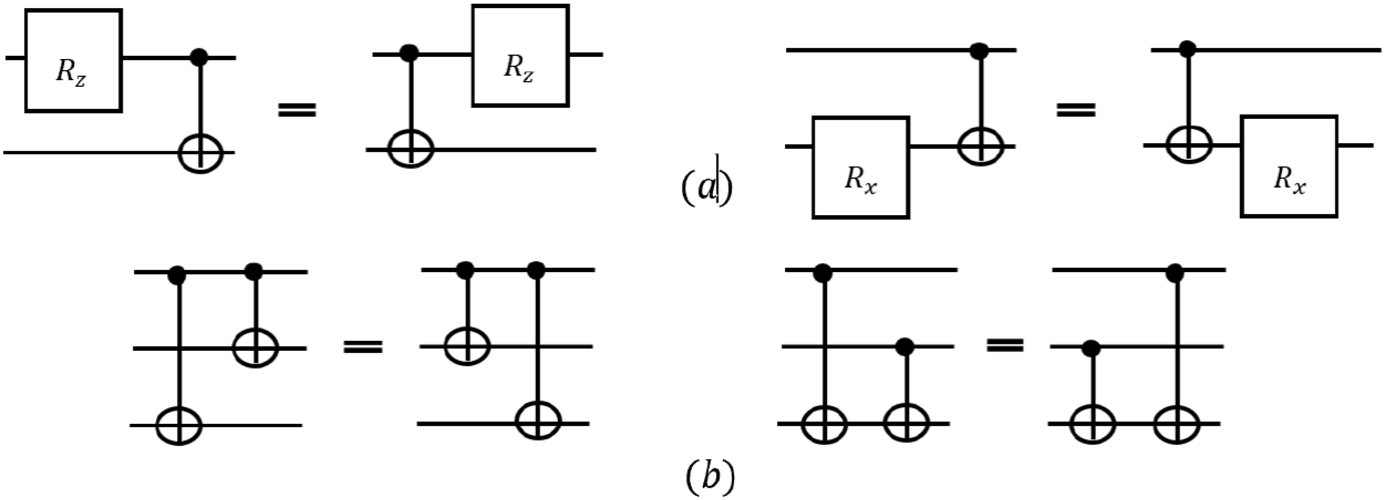
(**a**) Commuting of the rotation gates and the CNOT gate. (**b**) Commuting of two CNOT gates in two different circumstances^38^.

In some cases, the gates that can be merged or removed are not placed side by side. By moving the gates according to Figs. 4 and 5, the gates are placed side by side and so they can be merged and removed by the above rules. . For this purpose, the Sympy library^39^ is used. Sympy is an open source Python library for symbolic mathematics. With the help of this library, complex quantum circuits can be transformed into simple ones. One of the interesting features of this library is producing equivalent circuits. In fact, using library different gates in the circuit are moved around and all locations where the gates can be placed are examined. Then, the rules 1-4 are re-examined by the algorithm and the circuit is simplified if conditions pass.

**Figure 6:**
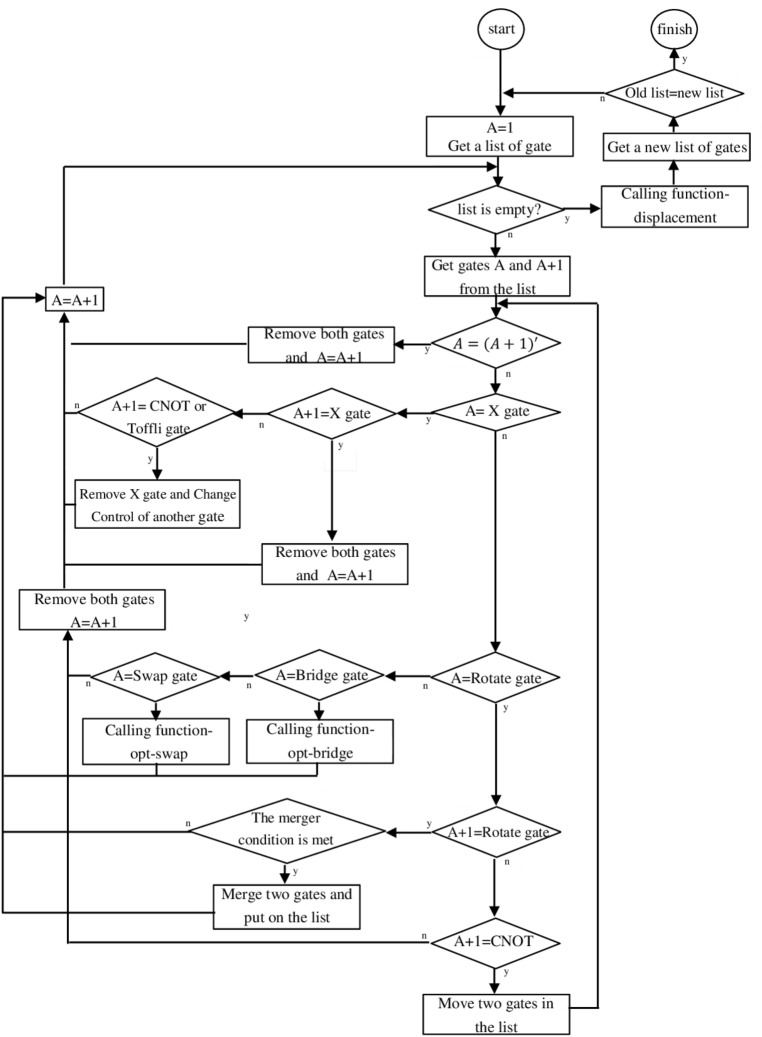
The flowchart of the steps of the optimization approach using rules 1–4.

**Figure 7:**
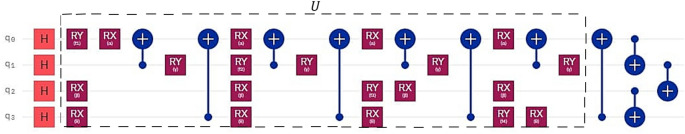
A quantum circuit with a quantum transfer learning method^28^.

These defined rules are applied in a loop until no further improvement is obtained. Algorithm 1 and Fig. 6 present the steps of our optimization approach using the above rules. Using this framework, we optimized the quantum machine learning circuit of a classification task for medical diagnosis using quantum transfer learning^28^. This circuit has been tested in several real quantum processors as well as various simulators. This quantum circuit aims at distinguishing a sick person from a healthy person based on computed tomography images. The circuit consists of four steps: The Hadamard gate is first applied to all qubits and then with the help of U operator defined in^28^, the classical data is encoded and then entanglement is created. The dotted box of Fig. 7 shows one application of this operator. Finally, the qubits are measured. Figure 7 demonstrates the quantum circuit of this quantum machine learning algorithm with only one repetition of the sub-circuit.

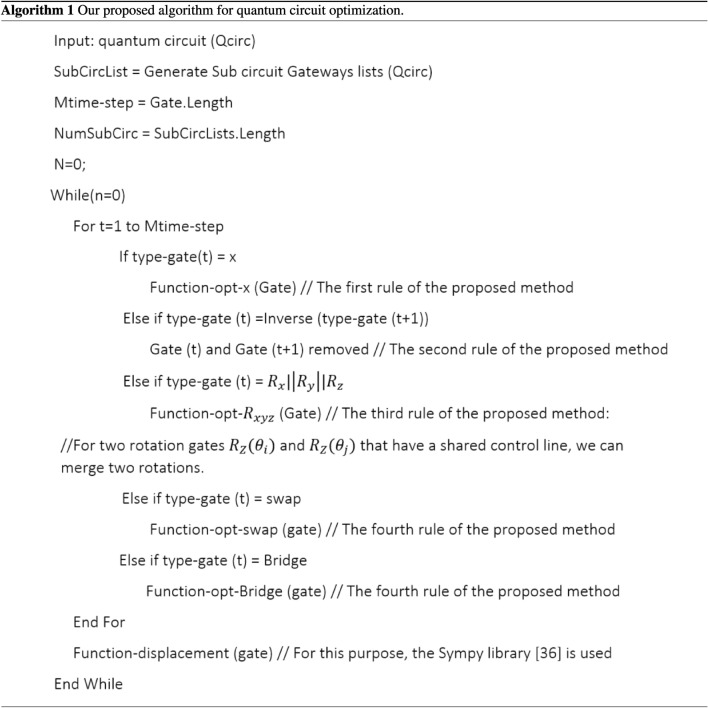



## Results and discussion

In this section, the experimental results for different quantum circuits and quantum machine learning circuits are presented. The file of these circuits are mostly in QASM format, received as input by Python language and then converted into a matrix by the Qiskit library of Python^46^. Then the proposed optimization techniques are applied to them. The main criterion in evaluating the proposed method is the comparison of the number of gates before and after optimization. Also, another criterion we considered in this work is the execution time of the quantum circuits and the amount of time step reduction. Qiskit library was used in IBM Q quantum computers for output verification. The optimization is then performed and the circuit resulting from the optimization was given to the simulator and the number of gates is calculated after the simulation. Obviously, in order to obtain the simulation results, the pre-optimization and post-optimization simulations must be the same. As the number of gates decreases from the initial value after applying the optimization model, the speed of the algorithm and its implementation cost improve. In this case, the proposed model will be a more efficient model. In order to verify our approach, we first tested our approach on different general quantum circuits and the results are shown in Table 1. In this table, each column is the corresponding quantum circuit and for each circuit we showed the improvement caused by our optimization approach.

**Table 1:** Results of implementation of the proposed method on different quantum circuits.

Quantum circuit	#of qubits	Pre-optimization			After Optimization			Improvement(%)			Speed up	References
		Execusion time Imp. (S)	#of Time steps	#of Gates	Execution time Imp. (S)	#of Time steps	#of Gates	Execution time Imp. (S)	#of Time steps	#of Gates		
VQC	3	6.4	13	21	6.3	13	18	1.56	0	14.28	0.1	^40^
Q transfer learning	4	6.8	13	28	6.2	13	25	8.82	–	10.71	0.6	^29^
Q neural network	10	6.9	17	67	6.6	16	57	4.34	5.88	14.92f	0.3	^28^
Grover 1	4	6.4	26	41	6.2	16	21	3.12	38.46	48.78	0.2	^41^
Grover 2	4	6.7	21	39	5.9	17	27	11.94	19.09	30.76	0.8	^41^
QAOA	2	6.7	7	11	6.2	5	7	7.46	28.57	36.36	0.5	^42^
test circuit K Means	3	6.5	14	26	6.2	14	24	4.6	0	7.7	0.3	^43^
TNN	4	6	10	27	5.9	8	14	1.7	20	48.14	0.1	^44^
KNN	4	7.8	20	33	4.7	8	13	39.74	60	60.60	3.1	^45^

**Table 2:** Comparison results of the proposed method with state-of-the-art optimization methods on different quantum circuits.

Circuit name	Method	2-qubit gate count	Depth	Time
Graycode6-47	ZX Calculus	5	5	0.08
	Quilc	5	5	1.03
	tket	5	5	0.06
	AQCEL	5	5	2.08
	Proposed method	5	5	0.04
4gt11-84	ZX Caculus	8	10	0.76
	Quilc	8	7	0.83
	tket	12	11	0.01
	AQCEL	8	7	0.98
	Proposed method	8	7	0.06
4mod-v1-24	ZX Calculus	15	28	1.53
	Quilc	18	17	1.64
	tket	26	25	0.05
	AQCEL	19	22	0.87
	Proposed method	28	16	0.03
Decod24-bdd-294	ZX Calculus	42	48	1.43
	Quilc	46	36	2.16
	tket	59	52	0.04
	AQCEL	45	46	2.87
	Proposed method	54	38	0.02
Mini-alu-305	ZX Calculus	112	110	4.37
	Quilc	135	78	16.32
	tket	178	112	0.09
	AQCEL	172	95	0.32
	Proposed method	168	90	0.05
PF4_30_after	ZX Calculus	23386	18380	215.06
	Quilc	3632	2822	139.35
	tket	3614	2798	5.33
	Proposed method	3638	2798	4.86
Dc2_222	ZX Calculus	3426	3620	145.15
	Quilc	4130	3517	113.25
	tket	4130	3517	2.83
	Proposed method	4131	3517	1.83
Square_root_7	ZX Calculus	2295	2052	90.47
	Quilc	2560	2102	2.64
	tket	3089	2102	2.56
	Proposed mthod	2698	2098	1.43
Pf4_20_before	ZX Calculus	3044	2168	56.08
	Quilc	1182	1182	76.33
	tket	1182	1182	1.52
	Proposed Method	1196	1194	1.39

Also, in Table 2 the comparison between our proposed approach and other works in the literature ZX-calculus^50^, AQCEL^51^, tket^52^, and Quilc^53^ are presented. It can be seen from this table that our approach works better in terms of circuit depth compared to other approaches and also in many circuits it is better in terms of the number of 2-qubit gates, while the execution time of our method is better than all other approaches.

**Figure 8:**
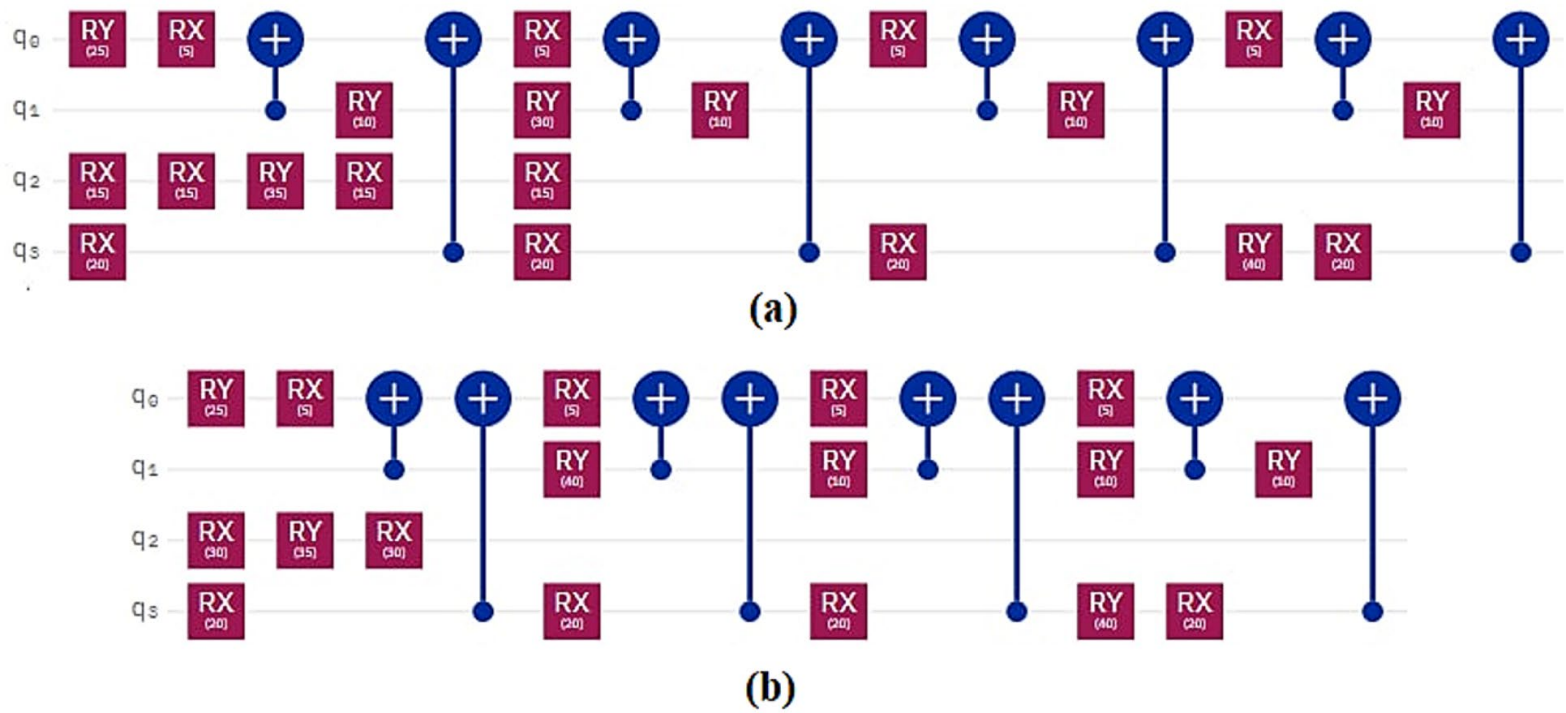
Demonstration of original and improved quantum transfer learning circuits. Diagram (**a**) shows the non-optimal circuit and diagram (**b**) shows the improved circuit with reducing the number of gates by 10.7%.

**Figure 9:**
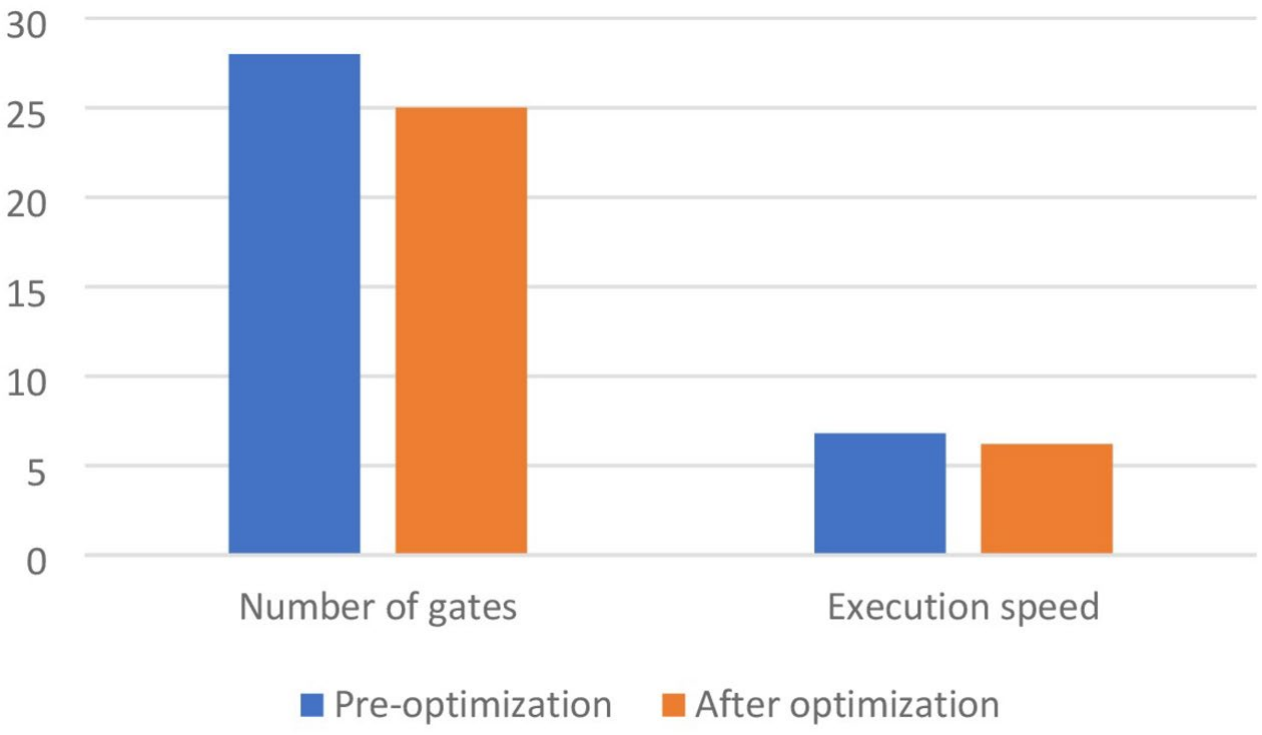
Demonstration of optimized and non-optimized diagrams of quantum transfer learning circuits.

In the proposed method, assuming that the number of time steps is *N* and the number of qubits is *Q*, the time complexity of the algorithm is obtained as *O*(*NQ*). As shown in Tables 1 and 2 applying our method to a variety of quantum circuits reduces the number of gates, time steps, and execution time of the quantum circuits significantly. At the second part of the experiments, our optimization approach was applied on the quantum machine learning circuits. One of these circuits uses transmission learning method for a potential application in medical diagnosis. By applying the proposed method to the above quantum circuit, only the U-shaped part of the circuit improves as shown in Fig. 8. In Fig. 8a it can be seen that the original circuit from^28^ has 28 quantum gates. Figure 8b shows the improved circuit diagram with 10.7% reduction in the number of gates. This is the amount of quantum cost reduction for one repetition below the U-circuit in the main circuit. For cases where this sub-circuit is repeated many times in the main circuit, the rate of improvement increases. In this case, by applying the proposed method on circuits with big data, desirable results will be obtained. The results of the implementation of the proposed method on the quantum circuit of transfer learning are shown in Fig. 9 before and after optimization.

**Figure 10:**
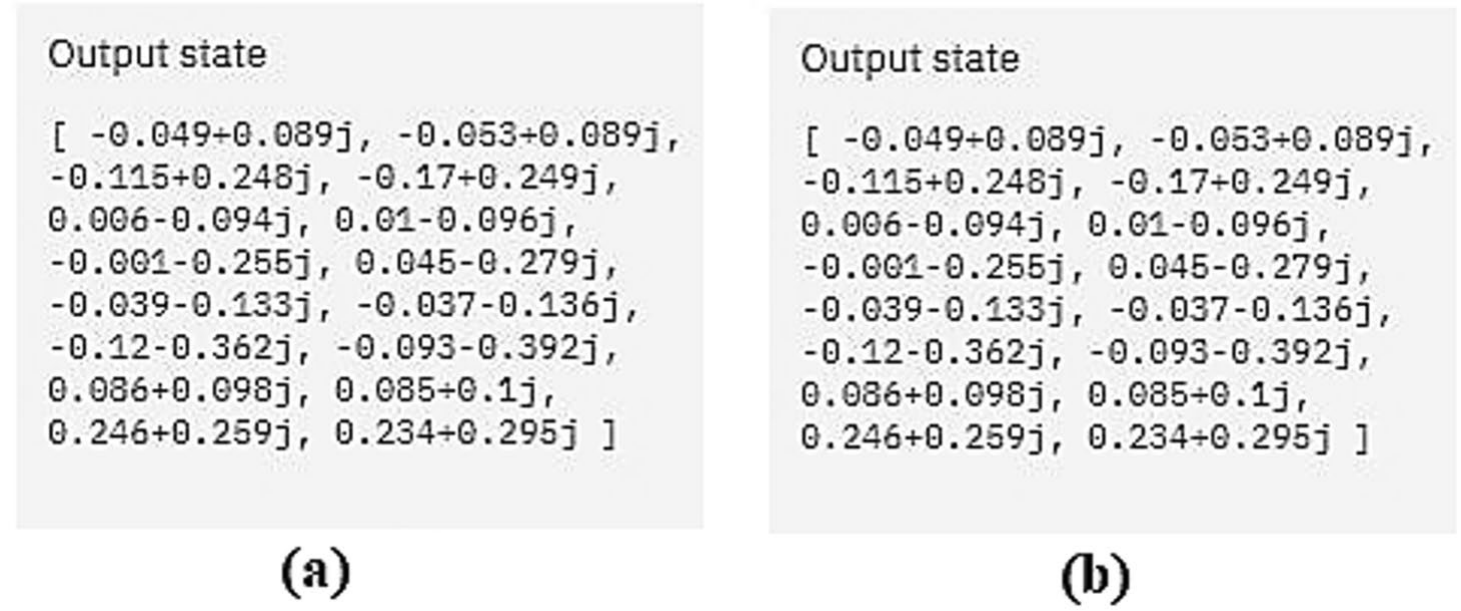
The simulation result of the output of the quantum transfer learning circuit before and after optimization in (**a**) and (**b**), respectively. The results are identical.

We verified the outputs in IBM Q and the results are demonstrated in Fig. 10. Figure 10a is the output of the original circuit and Fig. 10b is the output after we applied our optimization algorithm. Since the output is the same in both cases, the transformation has done correctly.

**Figure 11:**
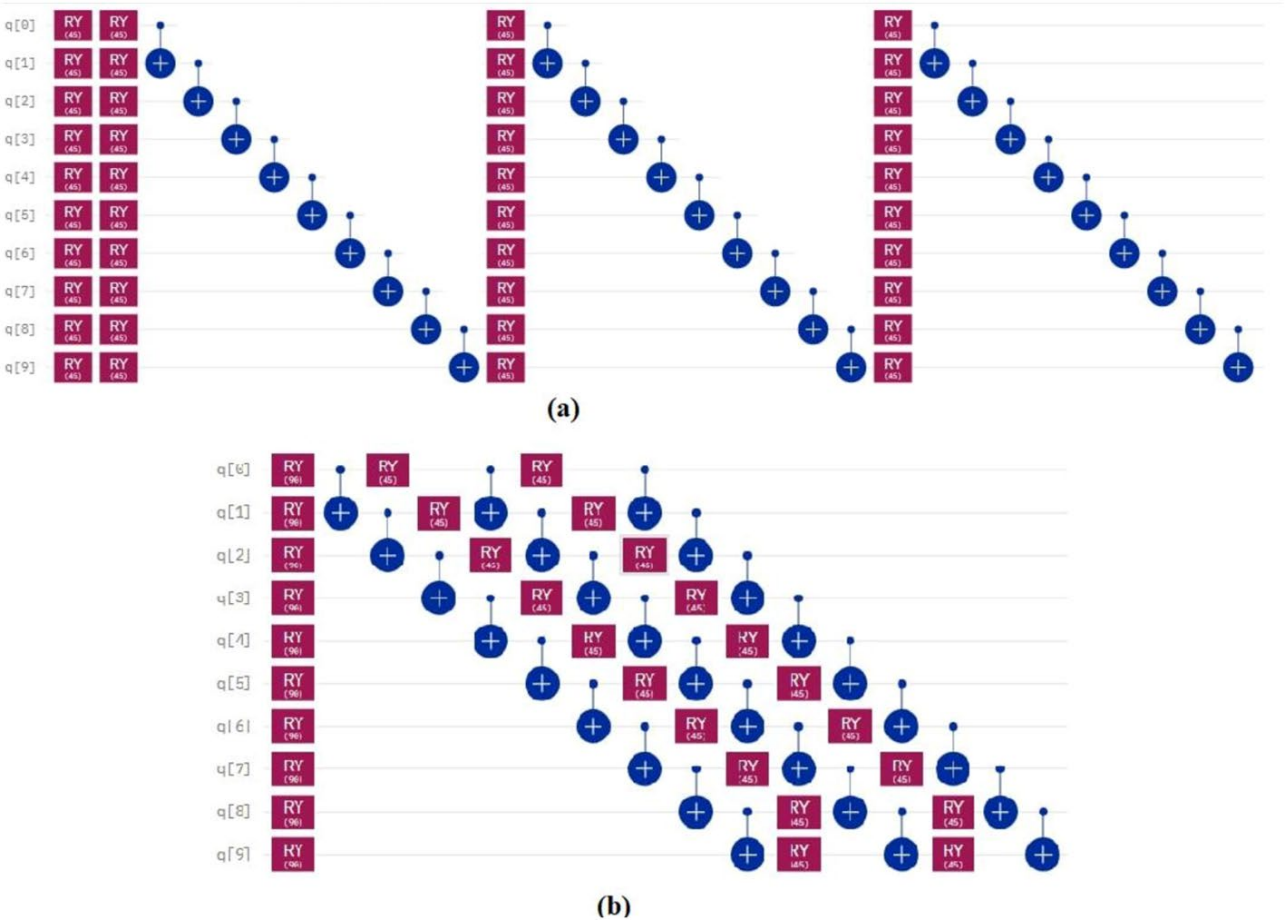
Demonstration of original^30^, and (**b**) improved quantum neural network circuits used for cancer diagnosis. The non-optimal circuit (**a**) is executed in 17 time steps, but the improved circuit, which has a reduction of 14.9% in the number of gates, its time step is reduced to 16.

**Figure 12:**
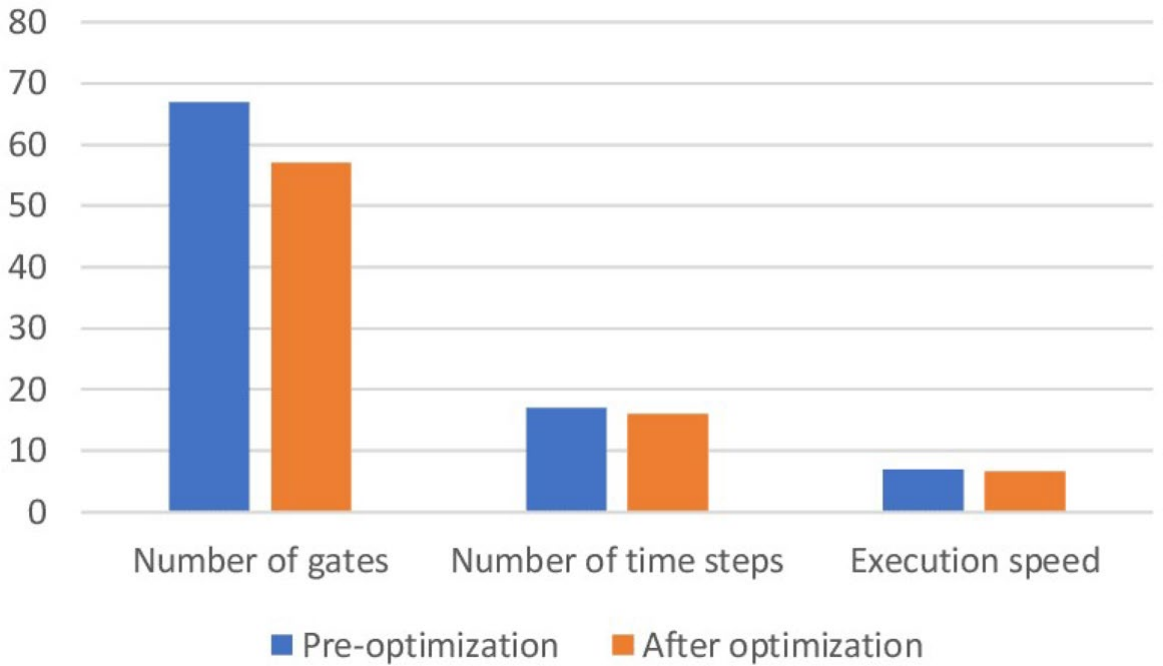
Comparison between optimized and non-optimized quantum neural networks circuits.

The next quantum machine learning circuit that we used in this work is the quantum circuit of the neural network for cancer detection^30^ which used the design and operation of a classical neural network but it is a quantum neural network capable of working on a 10 qubit system. By demonstrating network performance, the authors have tried to use the basic principles of machine learning to manage data. The graphical representation of this circuit is shown in Fig. 11. Figure 11a shows the original circuit from^30^ which is implemented in 17 time steps with 67 quantum gates. Figure 11b shows the improved circuit which in addition to a 14.9% reduction in the number of gates, reduces its time steps to 16. The comparison result of applying the proposed method to this circuit is shown in Fig. 12.

**Figure 13:**
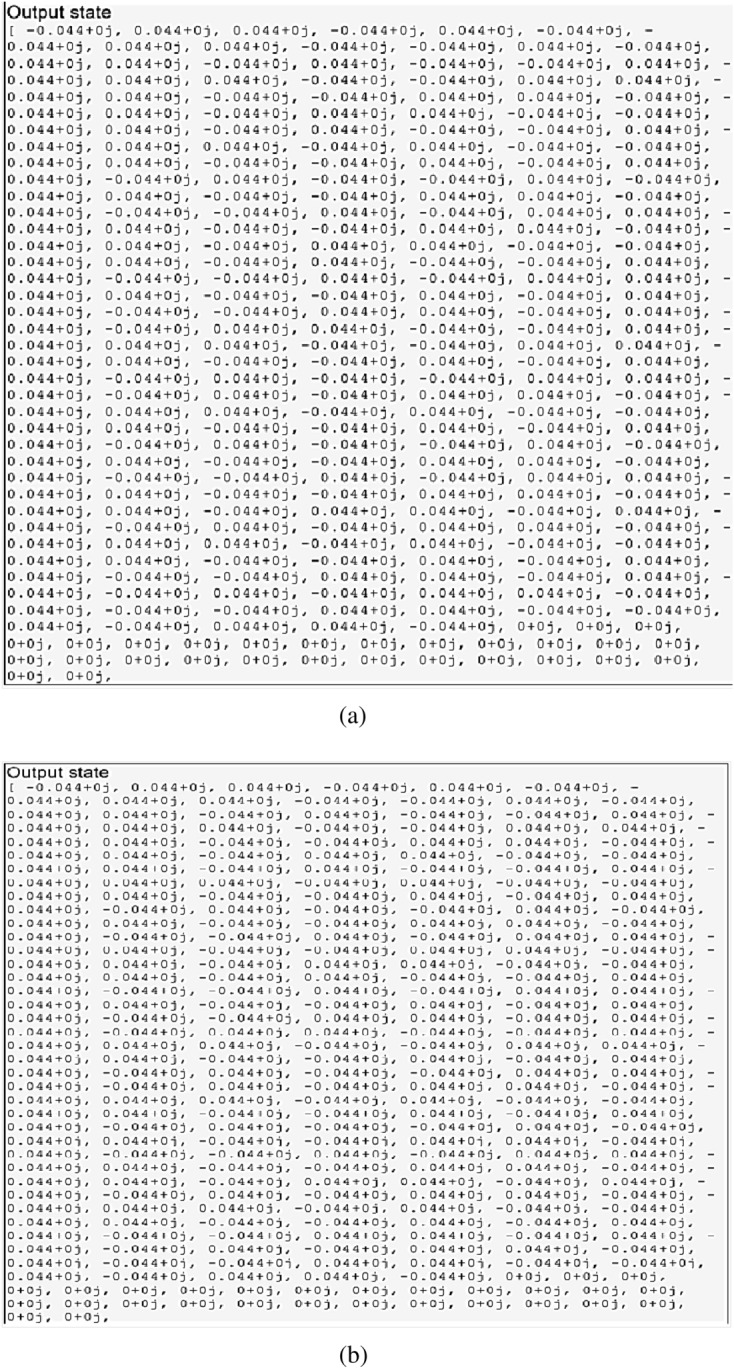
The simulation result of the output of the quantum neural network circuit before and after optimization in (**a**) and (**b**) respectively. The results are identical.

The output results of the circuits are shown in Fig. 13. Figure 13a is the output of the original circuit and Fig. 13b is the output after we applied our optimization algorithm. Since the output is the same in both cases, it shows that the transformation of the proposed optimization is correct.

**Figure 14:**
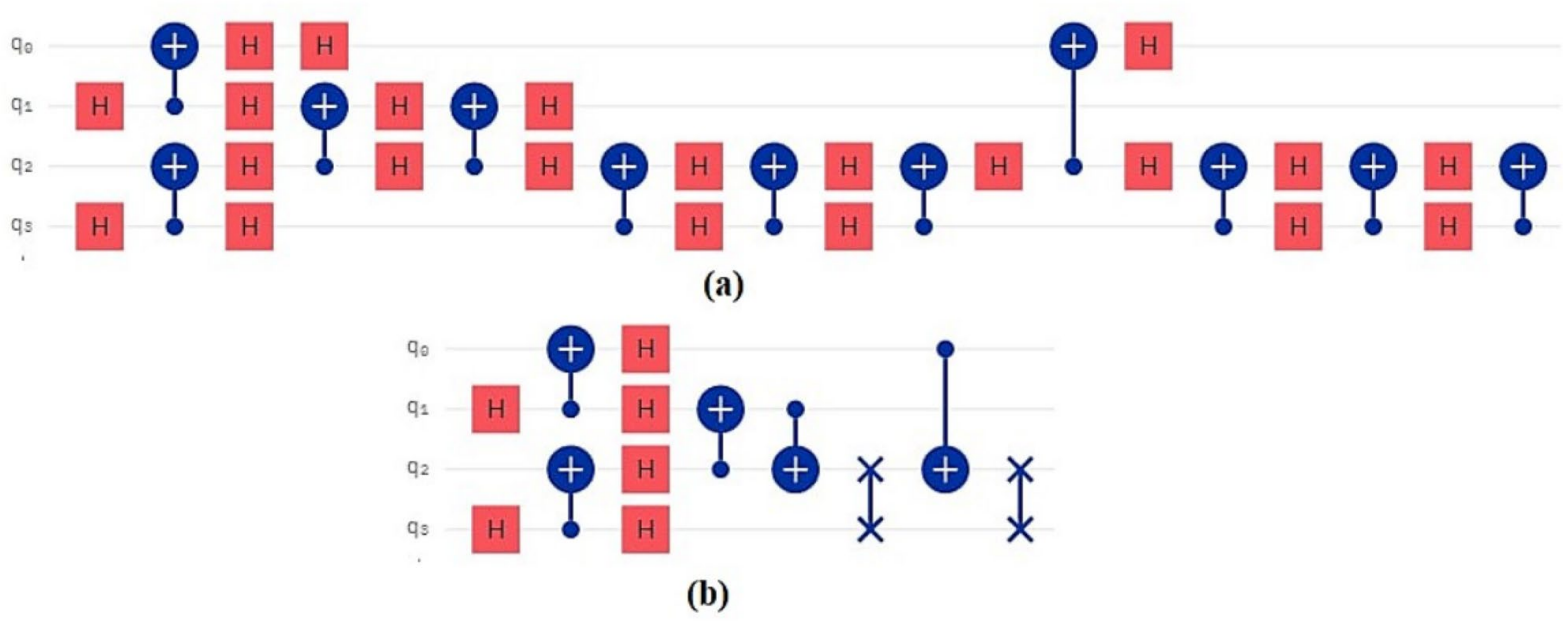
(**a**) Demonstration of original^30^, and (**b**) improved quantum circuits test for the KNN algorithm. The non-optimal circuit (a) is executed in 20 time steps, but the improved circuit, which has a reduction of 60.60% in the number of gates, its time step is reduced to 8.

**Figure 15:**
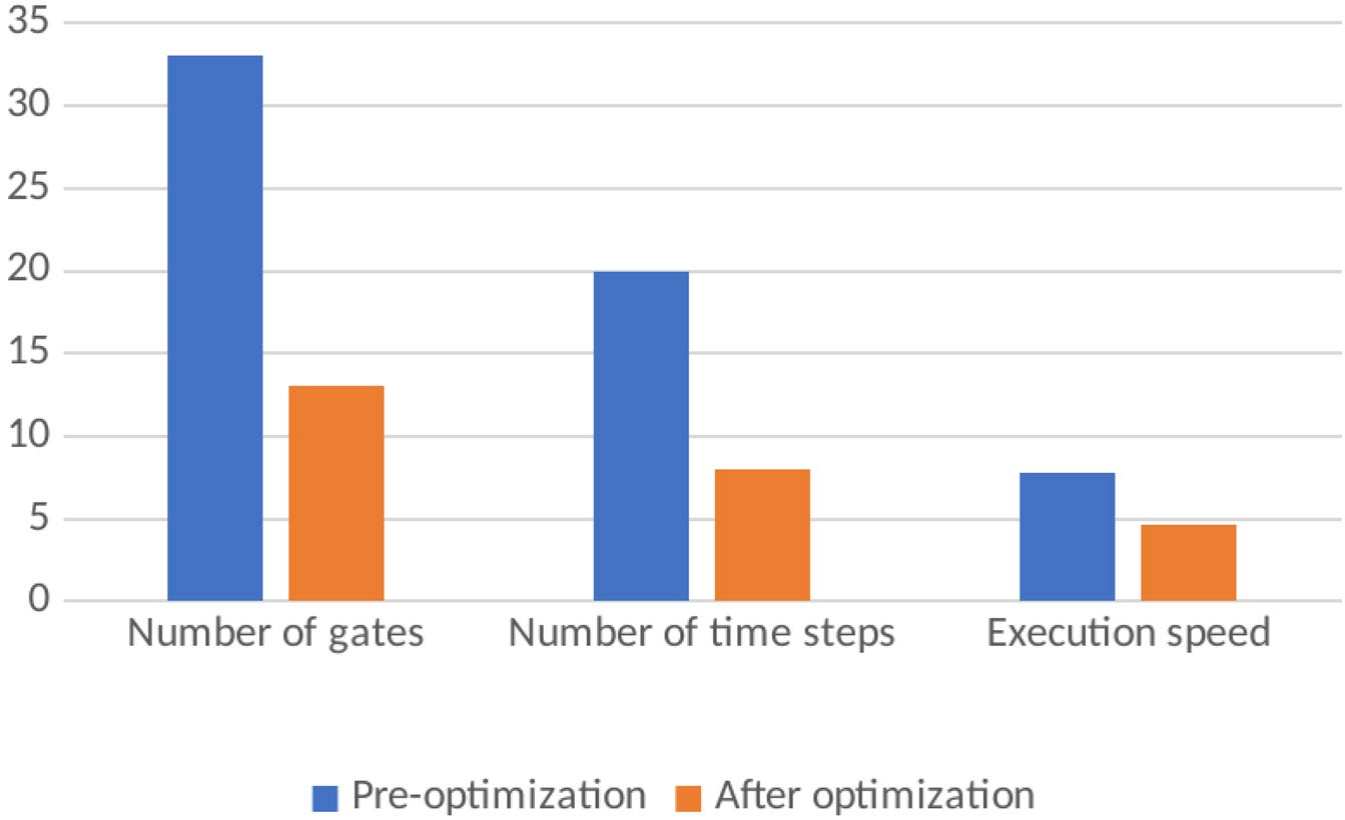
Comparison between optimized and non-optimized quantum circuits test for the KNN algorithm.

The next quantum machine learning circuit that we used in this work is Fig. 14. The quantum repeater circuit is used as a test for the KNN algorithm in^45^. The graphical representation of this circuit is shown in Fig. 14. Figure 14a shows the original circuit, which is implemented in 20 time steps with 33 quantum gates. Figure 14b shows the improved circuit, which in addition to a 60.60% reduction in the number of gates, reduces its time steps to 8. The comparison result of applying the proposed method to this circuit is shown in Fig. 15.

**Figure 16:**
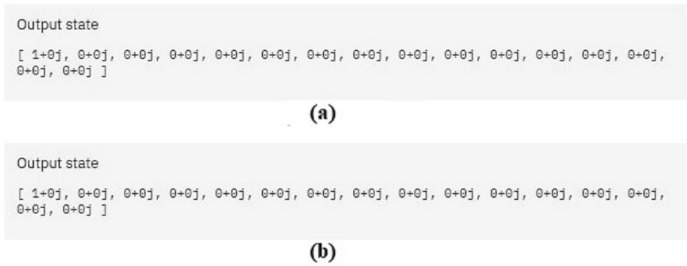
(**a**) The simulation result of the output of the quantum circuits test for the KNN algorithm before and after optimization in (**a**) and (**b**), respectively. The results are identical.

We verified the outputs in IBM Q and the results are demonstrated in Fig. 16. Figure 16a is the output of the original circuit and Fig. 16b is the output after we applied our optimization algorithm. Since the output is the same in both cases, the transformation has done correctly.

## Conclusion

Realizing machine learning algorithms in a quantum system for big data is a real challenge but with remarkable advantages of using quantum computers. In quantum circuits, as the number of gates increases, the number of time steps and execution time is also increased, which is why optimizing quantum circuits is an effective way to overcome these problems. In this study, a new general framework of quantum circuit optimization was presented and in particular, quantum machine learning algorithms for big data were investigated in order to improve their quantum circuit model which in turn leads to the improvement and reduction in the number of required quantum computation resources. In fact, by applying the proposed method, quantum circuits were implemented in less time than the original circuits, with the same functionality of the original design. In addition, applying this method also reduces the quantum costs. Several quantum circuits with different functionality and algorithms were used to evaluate the proposed method. The results of the improved circuits showed that the number of quantum gate, the time steps, and the execution time in the evaluated circuits were reduced. In particular, the proposed method was investigated on the quantum circuits of transfer learning and neural network. Our approach reduced the number of the gates by 10.71% respectively in transfer learning circuit and also reduced the number of time steps and the gate by 27.2% and 14.9% respectively in neural network circuit. More importantly, this was the amount of reduction for one iteration of the U-subcircuit in the main circuit of the transfer learning algorithm. So, for the cases where this sub-circuit was repeated more often in the main circuit, the optimization is even more. So, by applying the proposed method on circuits with big data, better results would be obtained.

## Data Availability

The datasets used and/or analysed during the current study available from the corresponding author on reasonable request.
